# Supplementary material for: Role of Advanced Glycation End Products as New Biomarkers in Systemic Lupus Erythematosus
Source: Int J Mol Sci. 2024 Mar 5;25(5):3022. doi: 10.3390/ijms25053022 (PMC10931748; doi:10.3390/ijms25053022)
Supplement: Supplementary file 1 [file ijms-25-03022-s001.zip › ijms-2867865-supplementary.pdf]

# Supplementary Materials

Recorded variables and their classifications.

- Demographics like age, gender, and ethnicity.
- Disease characteristics: All of the criteria gathered in the 1997 ACR and 2012 SLICC SLE classificatory criteria were recorded, as well as other characteristics such as different autoantibodies or overlap with other autoimmune diseases.
    - Disease duration was divided into 4 groups: 0-5, 6-10, 11-20 and ≥ 20 years since SLE diagnosis.
    - Leukocyturia and hematuria were divided into 5 categories according to the amount of leukocytes/red blood cells detected per field (S0: none, S1: 0-5, S2: 5-10, S3: 10-20, S4: 20-50, S5: >50/camp).
- Treatment variables: all specific treatments for SLE patients were receiving at the moment of inclusion were recorded. We created three groups with treatment regimens progressively more intense: patients receiving no treatment or only corticosteroids vs patients receiving also antimalarials ± corticosteroids vs patients receiving immunosuppressants/biological drugs ± antimalarials ± corticosteroids. We also recorded treatments for CVRF like antihypertensive or antidyslipemic drugs, antiplatelet or anticoagulant therapy.
- Different indexes for measuring SLE activity and accrual damage
    - Physician global assessment (PGA): divided into <1, 1-2, >3 according to the distribution in the sample.
    - Patient global assessment (PtGA) ≤ 3 vs >3, categorized according to the nonlinear association observed in the scatter plot (see Supplementary Figure 3).
    - Disease Activity Score 28 (DAS28): remission ≤ 2.6, low activity >2.6-3.2, moderate activity >3.2-5.1, high activity >5.1.
    - SLE disease activity index (SLEDAI): remission=0, mild >0- ≤ 4, moderate >4-11, severe >11. For statistical purposes we grouped patients in remission and with mild activity.
    - SLICC/American College of Rheumatology (SLICC/ACR) Damage Index (SDI), analyzed both as a quantitative variable, categorized in two groups (SDI 0 vs ≥ 1), and in three groups (0 vs 1 vs ≥ 1).
- PROs like the Health Assessment Questionnaire (HAQ) (divided into normal<0.3, mild 0.3-1.3, moderate >1.3-1.8, severe >1.8), the Functional Assessment of Chronic Illness Therapy – Fatigue Scale (FACIT), and patient global assessment by a visual analogic scale (PtGA).
- Cardiovascular variables:
    - CVRF: indicates the presence of one of the following: obesity (BMI > 30 Kg/m$^2$), AHT, DLP, CRD or hyperuricemia. The smoking status, which was considered as a separate variable due to its high correlation with AGEs levels.
    - Cardiovascular events (CVE): angina, myocardial infarction, cerebrovascular accident, peripheral arterial disease, intestinal ischemia or ischemia of some other territory). CVRF&CVE indicates the presence of either/both CVRF or CVE (CVRF&CVE>0) or the sum (CVRF&CVE).

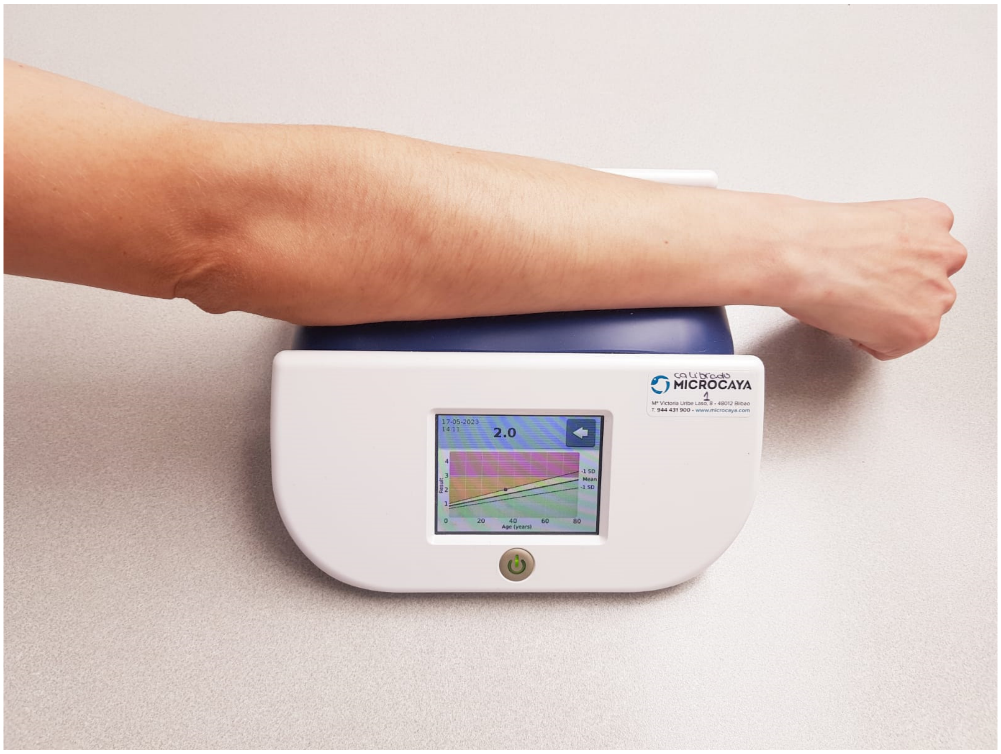


**Supplementary Figure S1.** Advanced glycation end products (AGEs) reader and how they are measured in the volar side of the forearm of subjects.

**Supplementary Table S1.** Other demographic and disease characteristics of SLE patients and their distribution according to AGEs tertiles in the bivariate analysis. M1: adjusted by age, M2 adjusted by age and smoking. "c" indicates variables which have been categorized as previously stated in the methodology section. Bold indicates p-value <0.1 and * indicates values according to the blood test performed in the study. *ANA: antinuclear antibodies; DAS28: Disease activity score 28; HAQ: Health Assessment Questionnaire Disability Index; VAS: Visual analogic scale; ESR: Erythrocyte sedimentation rate; CH50 and C3: Complement CH50 and C3; APS: antiphospholipid syndrome; SADs: systemic autoimmune diseases; CVRF: cardiovascular risk factors (obesity = IMC> 30 Kg/m2, arterial hypertension, dyslipidemia, chronic renal disease or hyperuricaemia); CVE: cardiovascular events (angina, myocardial infarction, cerebrovascular accident, peripheral arterial disease, intestinal ischemia or ischemia of some other territory);cDMARD; Disease-modifying antirheumatic drugs; bDMARD: biological DMARD.*

| **Variables** | **All** | **1st tertile [1.2,2.3)** | **2nd tertile [2.3,2.8)** | **3rd tertile [2.8,4.6]** | **p-val M1** | **p-val M2** |
|---|---|---|---|---|---|---|
| | N=122 | N=44 | N=41 | N=37 | | |
| Gender: Female | 114 (93.4%) | 40 (90.9%) | 38 (92.7%) | 36 (97.3%) | 0.429 | 0.333 |
| Body mass index | 25.4 (4.74) | 24.7 (5.00) | 24.9 (3.21) | 26.8 (5.58) | 0.341 | 0.132 |
| Ethnicity | | | | | 0.614 | 0.747 |
| Caucasian | 81 (66.4%) | 25 (56.8%) | 25 (61.0%) | 31 (83.8%) | | |
| Latin | 29 (23.8%) | 16 (36.4%) | 9 (22.0%) | 4 (10.8%) | | |
| Other | 12 (9.84%) | 3 (6.82%) | 7 (17.1%) | 2 (5.41%) | | |
| Classificatory Criteria and Other Clinical and Serological Data | | | | | | |
| Constitutional symptoms | 11 (9.02%) | 8 (18.2%) | 1 (2.44%) | 2 (5.41%) | 0.520 | 0.578 |
| Cutaneous | 91 (74.6%) | 32 (72.7%) | 28 (68.3%) | 31 (83.8%) | 0.348 | 0.361 |
| Photosensitivity | 74 (60.7%) | 25 (56.8%) | 27 (65.9%) | 22 (59.5%) | 0.446 | 0.402 |
| Alopecia | 55 (45.1%) | 22 (50.0%) | 15 (36.6%) | 18 (48.6%) | 0.715 | 0.963 |
| Serositis | 10 (8.20%) | 4 (9.09%) | 5 (12.2%) | 1 (2.70%) | 0.951 | 0.988 |
| Neurological | 11 (9.02%) | 5 (11.4%) | 2 (4.88%) | 4 (10.8%) | 0.974 | 0.853 |
| Hematological | 87 (71.3%) | 32 (72.7%) | 26 (63.4%) | 29 (78.4%) | 0.716 | 0.344 |
| ANA+ ever | 122 (100%) | 44 (100%) | 41 (100%) | 37 (100%) | | |
| Anti-dsDNA+ ever | 77 (63.1%) | 25 (56.8%) | 25 (61.0%) | 27 (73.0%) | 0.471 | 0.585 |
| Anti-Sm+ ever | 22 (18.0%) | 11 (25.0%) | 5 (12.2%) | 6 (16.2%) | 0.974 | 0.867 |
| Low complement | 64 (52.5%) | 25 (56.8%) | 24 (58.5%) | 15 (40.5%) | 0.733 | 0.540 |

| | | | | | | |
|---|---|---|---|---|---|---|
| Positive direct Coombs | 10 (14.1%) | 3 (12.0%) | 3 (13.6%) | 4 (16.7%) | 0.103 | 0.179 |
| APS antibodies carrier | 29 (23.8%) | 6 (13.6%) | 14 (34.1%) | 9 (24.3%) | 0.560 | 0.681 |
| APS | 5 (4.10%) | 2 (4.55%) | 2 (4.88%) | 1 (2.70%) | 0.553 | 0.288 |
| Other SADs | 77 (63.1%) | 33 (75.0%) | 20 (48.8%) | 24 (64.9%) | 0.223 | 0.315 |
| Anti-Ro60+ ever | 49 (40.2%) | 20 (45.5%) | 20 (48.8%) | 9 (24.3%) | 0.212 | 0.242 |
| Anti-Ro52+ ever | 28 (23.0%) | 11 (25.0%) | 9 (22.0%) | 8 (21.6%) | 0.542 | 0.482 |
| Pulmonary | 5 (4.10%) | 4 (9.09%) | 0 (0.00%) | 1 (2.70%) | 0.367 | 0.650 |
| Cardiac | 4 (3.28%) | 0 (0.00%) | 2 (4.88%) | 2 (5.41%) | 0.258 | 0.244 |
| Raynaud | 39 (32.2%) | 18 (40.9%) | 10 (24.4%) | 11 (30.6%) | 0.886 | 0.827 |
| ESR* | 11.0 [5.00;20.0] | 8.00 [2.50;15.0] | 11.0 [4.00;21.0] | 14.0 [7.50;20.2] | 0.271 | 0.213 |
| Anti-dsDNA+* | 4.00 [1.00;13.0] | 6.00 [1.00;16.5] | 3.00 [1.00;9.00] | 4.00 [1.00;14.0] | 0.443 | 0.538 |
| Anti-Ro52+* | 26 (21.8%) | 10 (23.8%) | 9 (22.5%) | 7 (18.9%) | 0.359 | 0.368 |
| CH50* | 60.3 [51.8;70.9] | 57.6 [44.1;68.9] | 62.7 [54.7;70.8] | 65.5 [54.7;72.1] | 0.806 | 0.779 |
| C3* | 106 (22.3) | 104 (25.3) | 103 (18.2) | 111 (22.6) | 0.254 | 0.164 |
| SLE Activity Indexes | | | | | | |
| cDAS28 | | | | | 0.668 | 0.606 |
| 0- Remission | 78 (65.0%) | 29 (67.4%) | 26 (63.4%) | 23 (63.9%) | | |
| 1- Low activity | 15 (12.5%) | 5 (11.6%) | 5 (12.2%) | 5 (13.9%) | | |
| 2- Moderate activity | 21 (17.5%) | 8 (18.6%) | 8 (19.5%) | 5 (13.9%) | | |
| 3- High activity | 6 (5.00%) | 1 (2.33%) | 2 (4.88%) | 3 (8.33%) | | |
| Patient Reported Outcomes | | | | | | |
| HAQ | 0.38 [0.00;0.88] | 0.31 [0.00;0.75] | 0.31 [0.00;0.91] | 0.38 [0.12;1.00] | 0.800 | 0.746 |
| Patient pain VAS | 2.00 [0.00;6.00] | 1.00 [0.00;5.00] | 4.00 [0.00;6.50] | 2.00 [0.00;5.00] | 0.660 | 0.607 |
| Comorbidities and Cardiovascular Disease | | | | | | |
| Hypertension | 26 (21.3%) | 3 (6.82%) | 10 (24.4%) | 13 (35.1%) | 0.946 | 0.673 |
| Dyslipidemia | 12 (9.84%) | 1 (2.27%) | 5 (12.2%) | 6 (16.2%) | 0.401 | 0.505 |
| Cardiovascular disease | 5 (4.10%) | 0 (0.00%) | 3 (7.32%) | 2 (5.41%) | 0.817 | 0.846 |
| Chronic renal disease | 3 (2.46%) | 1 (2.27%) | 1 (2.44%) | 1 (2.70%) | 0.331 | 0.364 |
| Hyperuricemia | 2 (1.64%) | 1 (2.27%) | 1 (2.44%) | 0 (0.00%) | 0.616 | 0.805 |
| Obesity | 22 (18.0%) | 8 (18.2%) | 3 (7.32%) | 11 (29.7%) | 0.746 | 0.505 |
| CVRF >0 | 47 (38.5%) | 11 (25.0%) | 16 (39.0%) | 20 (54.1%) | 0.465 | 0.592 |
| cCVRF | | | | | 0.498 | 0.703 |
| 0 | 75 (61.5%) | 33 (75.0%) | 25 (61.0%) | 17 (45.9%) | | |
| 1 | 33 (27.0%) | 8 (18.2%) | 13 (31.7%) | 12 (32.4%) | | |
| >1 | 14 (11.5%) | 3 (6.82%) | 3 (7.32%) | 8 (21.6%) | | |
| CVE | 9 (7.38%) | 3 (6.82%) | 3 (7.32%) | 3 (8.11%) | 0.216 | 0.095 |
| CVRF&CVE>0 | 48 (39.3%) | 12 (27.3%) | 16 (39.0%) | 20 (54.1%) | 0.495 | 0.590 |
| CVRF&CVE | | | | | 0.869 | 0.840 |
| 2 | 74 (60.7%) | 32 (72.7%) | 25 (61.0%) | 17 (45.9%) | | |
| 3 | 27 (22.1%) | 7 (15.9%) | 11 (26.8%) | 9 (24.3%) | | |
| 4 | 13 (10.7%) | 4 (9.09%) | 2 (4.88%) | 7 (18.9%) | | |
| 5 | 8 (6.56%) | 1 (2.27%) | 3 (7.32%) | 4 (10.8%) | | |
| Treatments | | | | | | |
| Antidyslipidemics | 14 (11.5%) | 2 (4.55%) | 6 (14.6%) | 6 (16.2%) | 0.729 | 0.942 |
| Antihypertensives | 28 (23.0%) | 4 (9.09%) | 10 (24.4%) | 14 (37.8%) | 0.970 | 0.758 |
| Antimalarials | 93 (76.2%) | 34 (77.3%) | 33 (80.5%) | 26 (70.3%) | 0.211 | 0.215 |
| cDMARD | 19 (15.6%) | 4 (9.09%) | 9 (22.0%) | 6 (16.2%) | 0.163 | 0.536 |
| bDMARD | 6 (4.92%) | 2 (4.55%) | 2 (4.88%) | 2 (5.41%) | 0.156 | 0.260 |
| Mycophenolate | 20 (16.4%) | 7 (15.9%) | 6 (14.6%) | 7 (18.9%) | 0.739 | 0.617 |
| Cyclosporine: 0 | 122 (100%) | 44 (100%) | 41 (100%) | 37 (100%) | | |
| Azathioprine | 19 (15.6%) | 6 (13.6%) | 8 (19.5%) | 5 (13.5%) | 0.396 | 0.263 |
| Cyclophosphamide: 0 | 122 (100%) | 44 (100%) | 41 (100%) | 37 (100%) | | |

**Supplementary Table S2.** Descriptive table of cases and healthy controls according to tertiles of AGEs and bivariate analysis. Bold indicates statistically significant differences. AGEs: advanced glycation end products; *HDL: High density lipoprotein; LDL: Low density lipoprotein.*

| **AGEs** | **[1.0,1.9)** | **[1.9,2.4)** | **[2.4,4.2]** | **Global p-value** | **p-value for trend** |
|---|---|---|---|---|---|
| | **N = 86** | **N = 87** | **N = 78** | | |
| Ethnicity | | | | 0.006 | 0.001 |
| Caucasian | 86 (100%) | 81 (93.1%) | 68 (87.2%) | | |
| Other | 0 (0.00%) | 6 (6.90%) | 10 (12.8%) | | |
| Age | 55.0 [51.0;60.0] | 56.0 [53.0;63.0] | 56.0 [53.0;61.8] | 0.112 | **0.090** |
| Sex | | | | 0.447 | 0.363 |
| Men | 5 (5.81%) | 6 (6.90%) | 2 (2.56%) | | |
| Women | 81 (94.2%) | 81 (93.1%) | 76 (97.4%) | | |
| Hypertension | 35 (40.7%) | 30 (34.5%) | 22 (28.2%) | 0.244 | **0.094** |
| Obesity | 24 (27.9%) | 30 (34.5%) | 19 (24.4%) | 0.344 | 0.646 |
| Dyslipidemia | 42 (48.8%) | 34 (39.1%) | 18 (23.1%) | **0.003** | **0.001** |
| Smoking | | | | **0.040** | 0.153 |
| Never | 34 (39.5%) | 46 (52.9%) | 23 (29.5%) | | |
| Former (>1 year) | 30 (34.9%) | 23 (26.4%) | 28 (35.9%) | | |
| Active | 22 (25.6%) | 18 (20.7%) | 27 (34.6%) | | |
| Body mass index | 28.5 (5.69) | 28.4 (6.30) | 27.2 (5.45) | 0.264 | 0.147 |
| Creatinine | 0.69 [0.59;0.77] | 0.71 [0.61;0.79] | 0.72 [0.64;0.84] | 0.135 | **0.046** |
| Uric acid | 4.85 (1.17) | 5.02 (1.49) | 4.68 (1.42) | 0.281 | 0.454 |
| Cholesterol | 214 (40.5) | 200 (34.6) | 199 (40.8) | **0.021** | **0.014** |
| HDL | 59.4 (12.5) | 63.9 (14.5) | 66.1 (16.0) | **0.079** | **0.027** |
| LDL | 142 (31.7) | 122 (28.3) | 121 (36.5) | **0.003** | **0.002** |
| Triglycerides | 134 [93.2;160] | 120 [96.0;157] | 94.5 [76.2;160] | **0.085** | **0.026** |
| Antidyslipidemics | 15 (17.4%) | 12 (13.8%) | 11 (14.1%) | 0.762 | 0.544 |
| Antihypertensives | 27 (31.4%) | 27 (31.0%) | 23 (29.5%) | 0.962 | 0.794 |

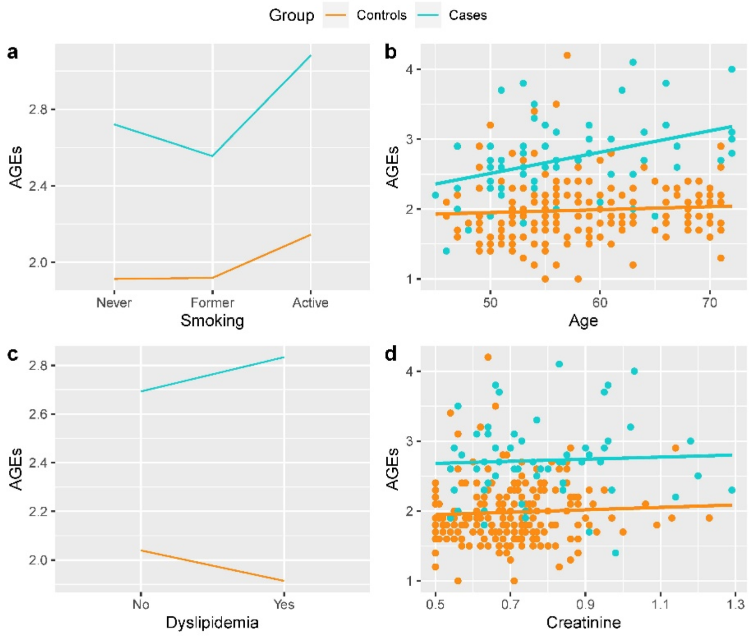


**Supplementary Figure S2.** Interaction graphs: a) Smoking; b) Age; c) Dyslipidemia; d) Creatinine.

**Supplementary Table S3.** Fixed-effects analysis of covariance (ANCOVA) model to study differences in dyslipidemia in the healthy controls group according to age and smoking status.

| | Est. | 2.5% | 97.5% | t val. | p-value |
|---|---|---|---|---|---|
| **(Intercept)** | 1.9282 | 1.8008 | 2.0555 | 29.8663 | <0.0001 |
| **Dyslipidemia (Yes)** | -0.1142 | -0.2415 | 0.0131 | -1.7701 | 0.0784 |
| **Age** | 0.0122 | 0.0014 | 0.0229 | 2.2308 | 0.0269 |
| **Smoking (Former and Never)** | 0.0583 | -0.1102 | 0.2269 | 0.6829 | 0.4955 |
| **Smoking (Active)** | 0.3023 | 0.1332 | 0.4714 | 3.5271 | 0.0005 |

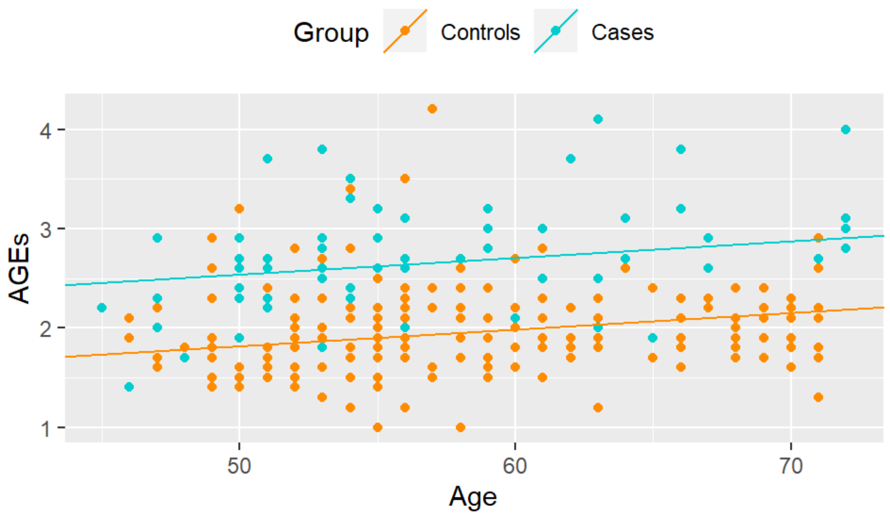


**Supplementary Figure S3.** Effects graphic of differences in AGEs values between cases and healthy controls according to age.

**Supplementary Table S4.** Statistically significant fixed-effects analysis of covariance (ANCOVA) model between AGEs and different varibles. SLEDAI: SLE disease activity index; SDI: SLE disease damage index (SDI); PGA: Physician global assessment (visual analogic scale 0-10; PtGA: Patient global assessment visual analogic scale 0-10; CRP: C-reactive protein levels in mg/dL; Leucocyturia defined as 0-5 according to number of leucocytes in urine per field; C4: Complement 4 levels (reference levels 10-40 mg/dL); IL-6 levels in pg/mL; Anti-Ro60+: Positivity of anti-Ro60 antibodies in the study blood test; ANA+: Positivity of anti-nuclear antibodies in the study blood test.

| Variable | | Est. | 2.5% | 97.5% | t val. | p-value |
|---|---|---|---|---|---|---|
| **SLEDAI: Remission/Mild** | **(Intercept)** | 2.3371 | 2.2066 | 2.4676 | 35.4786 | <0.0001 |
| | **cSLEDAI3: Moderate** | 0.2001 | 0.0006 | 0.3995 | 1.9867 | 0.0493 |
| | **cSLEDAI3: Severe** | 0.5188 | 0.1774 | 0.8601 | 3.0106 | 0.0032 |
| | **Age** | 0.0268 | 0.0204 | 0.0332 | 8.3580 | <0.0001 |
| | **Smoking (Yes)** | 0.3341 | 0.1168 | 0.5515 | 3.0452 | 0.0029 |
| **SDI [0-2]** | **(Intercept)** | 2.3158 | 2.1955 | 2.4361 | 38.1426 | <0.0001 |
| | **SDI [3,4]** | -0.0684 | -0.4372 | 0.3004 | -0.3673 | 0.7140 |
| | **SDI [5,6]** | 0.7169 | 0.1386 | 1.2951 | 2.4557 | 0.0156 |
| | **Age** | 0.0273 | 0.0211 | 0.0334 | 8.7537 | <0.0001 |
| | **Glucocorticoids (Yes)** | 0.3392 | 0.1250 | 0.5534 | 3.1362 | 0.0022 |
| | **Smoking (Yes)** | 0.4315 | 0.2227 | 0.6404 | 4.0937 | 0.0001 |
| **PGA < 1** | **(Intercept)** | 2.1256 | 1.8742 | 2.3770 | 16.7452 | <0.0001 |
| | **PGA [1,2]** | 0.3335 | 0.0580 | 0.6090 | 2.3975 | 0.0181 |
| | **PGA >2** | 0.3942 | 0.0943 | 0.6941 | 2.6031 | 0.0104 |
| | **Age** | 0.0291 | 0.0226 | 0.0357 | 8.7863 | <0.0001 |
| **PtGA ≤ 3** | **(Intercept)** | 2.4297 | 2.3092 | 2.5503 | 39.9056 | <0.0001 |
| | **PtGA>3** | 0.2622 | 0.0639 | 0.4605 | 2.6189 | 0.0100 |
| | **Age** | 0.0236 | 0.0172 | 0.0301 | 7.2499 | <0.0001 |

| Variable | | Est. | 2.5% | 97.5% | t val. | p-value |
|---|---|---|---|---|---|---|
| **CRP < 0.12** | **(Intercept)** | 2.3191 | 2.1560 | 2.4822 | 28.1631 | <0.0001 |
| | **CRP [0.12,0.28)** | 0.0925 | -0.1374 | 0.3224 | 0.7968 | 0.4272 |
| | **CRP [0.28,3.92]** | 0.2594 | 0.0359 | 0.4830 | 2.2988 | 0.0233 |
| | **Age** | 0.0267 | 0.0202 | 0.0332 | 8.1774 | <0.0001 |
| | **Smoking (Yes)** | 0.3627 | 0.1444 | 0.5809 | 3.2918 | 0.0013 |
| **Oral Ulcers** | **(Intercept)** | 2.4379 | 2.3124 | 2.5635 | 38.4462 | <0.0001 |
| | **Oral.Ulcers (Yes)** | 0.2162 | 0.0209 | 0.4116 | 2.1918 | 0.0304 |
| | **Age** | 0.0245 | 0.0180 | 0.0309 | 7.4832 | <0.0001 |
| **Leukocyturia 0** | **(Intercept)** | 2.3324 | 2.1981 | 2.4667 | 34.4129 | <0.0001 |
| | **Leukocyturia 1** | 0.1460 | -0.0958 | 0.3878 | 1.1960 | 0.2342 |
| | **Leukocyturia [2,5]** | 0.3695 | 0.1128 | 0.6261 | 2.8520 | 0.0052 |
| | **Age** | 0.0242 | 0.0174 | 0.0309 | 7.0873 | <0.0001 |
| | **Smoking (Yes)** | 0.3732 | 0.1566 | 0.5898 | 3.4131 | 0.0009 |
| **Anti-Ro60+** | **(Intercept)** | 2.6356 | 2.4858 | 2.7855 | 34.8364 | <0.0001 |
| | **Anti-Ro60 presence** | -0.2601 | -0.5027 | -0.0174 | -2.1227 | 0.0359 |
| **ANA+** | **(Intercept)** | 2.9889 | 2.5642 | 3.4136 | 13.9370 | <0.0001 |
| | **ANA presence** | -0.4961 | -0.9377 | -0.0545 | -2.2248 | 0.0280 |
| **C4 <18** | **(Intercept)** | 2.2585 | 2.0866 | 2.4305 | 26.0264 | <0.0001 |
| | **C4 [18,24)** | 0.2503 | 0.0200 | 0.4806 | 2.1530 | 0.0335 |
| | **C4 [24,49]** | 0.2854 | 0.0566 | 0.5143 | 2.4710 | 0.0150 |
| | **Age** | 0.0251 | 0.0182 | 0.0320 | 7.1965 | <0.0001 |
| | **Total cholesterol** | 0.0019 | -0.0008 | 0.0046 | 1.3890 | 0.1676 |
| | **Smoking (Yes)** | 0.3799 | 0.1554 | 0.6044 | 3.3533 | 0.0011 |
| **IL6** | **(Intercept)** | 2.2279 | 2.0384 | 2.4174 | 23.3132 | <0.0001 |
| | **cIL6 [1.88, 3.33)** | 0.1972 | -0.0521 | 0.4465 | 1.5691 | 0.1197 |
| | **cIL6 [3.33,144.10]** | 0.3524 | 0.0995 | 0.6053 | 2.7631 | 0.0068 |
| | **Age** | 0.0228 | 0.0155 | 0.0301 | 6.1716 | <0.0001 |
| | **Smoking (Yes)** | 0.3967 | 0.1678 | 0.6257 | 3.4370 | 0.0008 |

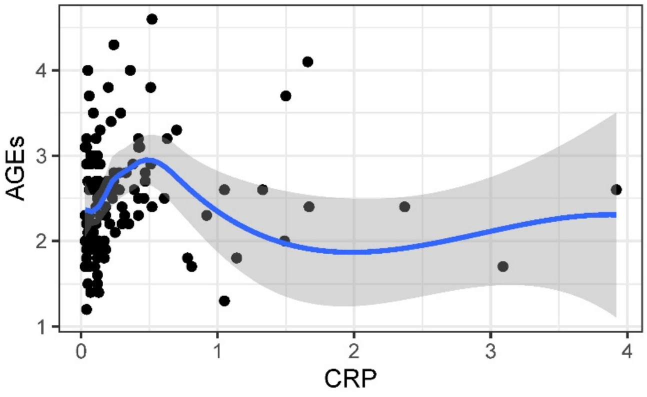


**Supplementary Figure S4.** C-reactive protein scatter plot.
